# Molecular cloning and preliminary function study of iron responsive element binding protein 1 gene from cypermethrin-resistant *Culex pipiens pallens*

**DOI:** 10.1186/1756-3305-4-215

**Published:** 2011-11-10

**Authors:** Wenbin Tan, Xiao Wang, Peng Cheng, Lijuan Liu, Haifang Wang, Maoqing Gong, Xin Quan, Honggang Gao, Changliang Zhu

**Affiliations:** 1 Department of Pathogenic Biology, Jining Medical University, Jining, Shandong Province, 272067, PR China; 2 Shandong Institute of Parasitic Diseases, A grand key laboratory of health department, Jining, Shandong Province, 272013, PR China; 3 Department of Pathogenic Biology, Nanjing Medical University; Key Laboratory of Modern Pathogenic Biology of Jiangsu Province, Nanjing, Jiangsu Province, 210029, PR China

## Abstract

**Background:**

Insecticide resistance jeopardizes the control of mosquito populations and mosquito-borne disease control, which creates a major public health concern. Two-dimensional electrophoresis identified one protein segment with high sequence homology to part of *Aedes aegypti *iron-responsive element binding protein (IRE-BP).

**Method:**

RT-PCR and RACE (rapid amplification of cDNA end) were used to clone a cDNA encoding full length IRE-BP 1. Real-time quantitative RT-PCR was used to evaluate the transcriptional level changes in the Cr-IRE strain *Aedes aegypti *compared to the susceptible strain of *Cx. pipiens pallens*. The expression profile of the gene was established in the mosquito life cycle. Methyl tritiated thymidine (^3^H-TdR) was used to observe the cypermethrin resistance changes in C6/36 cells containing the stably transfected IRE-BP 1 gene of *Cx. pipiens pallens*.

**Results:**

The complete sequence of iron responsive element binding protein 1 (IRE-BP 1) has been cloned from the cypermethrin-resistant strain of *Culex pipiens pallens *(Cr-IRE strain). Quantitative RT-PCR analysis indicated that the IRE-BP 1 transcription level was 6.7 times higher in the Cr-IRE strain than in the susceptible strain of 4th instar larvae. The IRE-BP 1 expression was also found to be consistently higher throughout the life cycle of the Cr-IRE strain. A protein of predicted size 109.4 kDa has been detected by Western blotting in IRE-BP 1-transfected mosquito C6/36 cells. These IRE-BP 1-transfected cells also showed enhanced cypermethrin resistance compared to null-transfected or plasmid vector-transfected cells as determined by ^3^H-TdR incorporation.

**Conclusion:**

IRE-BP 1 is expressed at higher levels in the Cr-IRE strain, and may confer some insecticide resistance in *Cx. pipiens pallens*.

## Background

Mosquitos are one of the medically important insects closely related to the life of human beings. The harm caused by mosquitoes to human beings is not only because of the harassment and blood feeding habits, but also in the transmission of various diseases, such as malaria [1], filariasis [2], yellow fever [3], dengue [4], and Japanese encephalitis [5]. It requires enormous efforts to overcome these diseases, which include environmental management, the use of insecticides and repellents, vaccine research and biological mosquito control [6-8]. Insecticides play a central role in controlling mosquitoes, but today, more and more serious insecticide resistance has appeared in mosquitoes, and against every chemical class of insecticides, such as Organochlorine, carbamate, organophosphate, pyrethroid and insect growth regulators [9-11]. Insecticide resistance jeopardizes the control of mosquito populations and mosquito-borne disease control, which creates a major public health concern [12-14].

Pyrethroids [15] are a group of chemicals that interact with insect ion channels causing a disruption to transmembrane potentials, therefore interfere with the normal functions of the insect nerve system. As a member of pyrethroids, cypermethrin [16] is commonly used for the treatment of bed nets and as a residual spray to help control mosquito population and disease transmission, such as malaria. Unfortunately the wide spread use and incorrect application of cypermethrin and other synthetic pyrethroids has accelerated the emergence of resistance in both targeted *Anopheles *species and off-target species such as *Cx. pipiens pallens *[17]. The elucidation of the resistance mechanism becomes important to guide the use of cypermethrin and the development of its substitutes.

To study the mechanism of cypermethrin resistance, a resistant strain of *Cx. pipiens pallens*, Cr-IRE, has been previously established in the laboratory of Shandong Institute of Parasitic Diseases by selecting stadium larvae with increasing concentrations of cypermethrin over 39 generations, and the cypermethrin-resistant level is 160.43 times higher than that of the sensitive strain [18]. Two-dimensional electrophoresis was used to screen differences in protein expression (Data not shown). One of the identified protein segments has high sequence homology to part of *Aedes aegypti *iron-responsive element binding protein (IRE-BP). Iron is necessary for a number of essential cell functions but, as excessive amounts can be toxic, iron metabolism is carefully controlled at both cellular and systemic levels. One of the iron homeostasis regulatory proteins is IRE-BP. IRE-BP is a cytosolic protein that binds a highly conserved sequence in the untranslated regions of mRNAs involved in iron metabolism including ferritin, IRE-BP 1 receptor, and erythroid 5-aminolevulinate acid synthase [19]. IRE-BPs has remained highly conserved during evolution. Iron-responsive elements (IREs) are regulatory RNA elements, which serve as specific binding sites for the IRE-binding protein (IRE-BP). Interaction between IREs and IRE-BP induces repression of ferritin mRNA translation and IRE-BP 1 receptor mRNA stabilization [20]. But the correlation between the function of IRE-BP 1 and insecticide resistance has not been reported to date.

In the present study, we utilized RT-PCR and RACE (rapid amplification of cDNA end) to clone a cDNA encoding full length IRE-BP 1. The nucleotide sequence of the clone was subsequently determined. Real-time quantitative RT-PCR indicated that this gene is transcribed to a greater extent in the Cr-IRE strain than in the susceptible strain of *Cx. pipiens pallens*. We also established the expression profile of the gene in the mosquito life cycle. Increased cypermethrin resistance in C6/36 cells containing the stably transfected IRE-BP 1 gene provided further support for a role of IRE-BP 1 in cypermethrin resistance in *Cx. pipiens pallens*.

## Materials and methods

### Mosquitoes

Cr-IRE strain and the susceptible strain of *Cx. pipiens pallens *mosquitoes used in this study were reared at 28°C with 70-80% humidity and a constant light:dark cycle (14:10). The mosquitoes were fed with mouse blood. The Cr-IRE colony has been selected from a susceptible strain [18], and the resistance has been maintained by treatment with cypermethrin at LC_50 _of each generation. The LC_50 _of Cr-IRE strain is 3.322 mg/L, 81-fold greater than that in the susceptible strain (0.041 mg/L).

### RNA extraction, 5'RACE PCR and RT-PCR

Total RNA was extracted from approximately 20 mg of 1st, 2nd, 3rd and 4th instar larvae and female adult *Cx. pipiens pallens *mosquitoes, using Trizol reagent (Gibco BRL, Grand Island, NY, USA) according to the manufacturer's protocol. The amplification of the 5' end of IRE-BP 1: forward primer: nested universal primer mix (UPM) was provided by the kit: (Primer 1, Table 1), reverse primer: designed based on the sequence reported by Saitoh, Y., [21] (GenBank Accession No. D16150.1): (Primer 2, Table 1). The amplification of the 3' end of IRE-BP 1: forward primer: designed based on the sequence reported by Saitoh, Y. [21] (GenBank Accession No. D16150.1): (Primer 3, Table 1), reverse primer: use the UPM primer proved by the 3'-RACE Kit: (Primer 4, Table 1). The cDNA fragments were successfully amplified and separated by electrophoresis on a 1% agarose gel. The cDNA fragment of interest was purified using a quick gel extraction kit (Qiagen, Hilden, Germany), and ligated into a pGEMT-easy vector (Tianwei, Beijing, China) for 5 min at 4°C. Plasmid DNA was extracted using a plasmid mini kit (Qiagen) and sequenced using the T7 promoter by Invitrogen Company (Applied Biosystems model 3730; Shanghai Invitrogen BioTech Company, Shanghai, China).

**Table 1:** Primers used in the experiments

Primer	Sequence
1	5'-AAGCAGTGGTATCAACGCAGAG-3'
2	5'-GAGCCTGGTGGAATAATCCTCAT-3'
3	5'-cactgacgtggaactcacttacT-3'
4	5'-CTAATACGACTCACTATAGGGC-3?
5	5'-atggctggtcctaacccctttca-3'
6	5'- TTAGGCAATCATCTTCCTGATCAT-3'
7	5'-GTGGCGAAGAGATGGATG-3'
8	5'-ACGAACAATAACCTGGAACT'
9	5'-AGCGTGAACTGACGGCTCTTG-3'
10	5'-ACTCGTCGTACTCCTGCTTGG-3'
11	5'-TACGGTGGTGCGGCTTAT-3'
12	5'-CAGGGTGAAATCTGATGGTT-3'
13	5'-atggctggtcctaacccctttca-3'
14	5'-GGCAATCATCTTCCTGATCATGT-3'
15	5'- atggctggtcctaacccctttca-3'
16	5'-GTTAGGGATAGGCT TACCTTCG-3'
17	5'-CACCAGGGTGTGATGGTCGG-3'
18	5'-CCACCGATCCAGACGGAGT-3'

After the 5' and 3' part of the sequence was obtained, a pair of forward primer (Primer 5, Table 1) and reverse primer (Primer 6, Table 1) was designed so that the full length cDNA of the gene could be obtained. RT-PCR conditions were: initial denaturation at 95°C for 5 min, followed by 32 cycles at 95°C for 1 min, 61°C for 1.5 min and 72°C for 2 min 30 s with a final 10-min extension at 72°C. The cDNA fragment obtained was ligated into a pGEMT-easy vector and sequenced. The NCBI database http://www.ncbi.nlm.nih.gov/BLAST/ was used to perform similarity searches and retrieve homologous sequences. The sequence analysis tools from the SWISS-PROT Internet server were used to process the data for the deduced protein sequences. Multiple sequence alignment was conducted using the DNAman software (version 4.13; Lynnon BioSoft, Quebec, Canada). The sequences included in our analysis for Sequence alignment and phylogenetic tree analysis were: *Aedes aegypti*: AY445078; *Canis familiaris*: XM_538698; *Citrus clementina*: FN552254; *Culex pipiens pallens*: HM443949; *Culex quinquefasciatus*: XM_001843334; *Danio rerio*: NM_001034983; *Drosophila melanogaster*: NM_058023; *Gallus gallus*: D16150; *Homo sapiens*: AF261088; *Manduca sexta*: AY032658; *Mus musculus*: AJ427344; *Pan troglodytes*: XM_001155874; *Salmo salar*: NM_001140230; *Tribolium castaneum*: XM_967008; *Tribolium castaneum*: XM_967008 and *Vitis vinifera*: XM_002263301.

### Real-time quantitative RT-PCR analysis

β-actin (forward: Primer 9, Table 1; reverse: Primer 10, Table 1). To confirm the accuracy and reproducibility of real-time quantitative RT-PCR, the experiment was determined in three repeats within one LightCycler run. The results for IRE-BP 1 were normalizβ-actin (forward: Primer 9, Table 1; reverse: Primer 10, Table 1). To confirm the accuracy and reproducibility of real-time quantitative RT-PCR, the experiment was determined in three repeats within one LightCycler run. The results for IRE-BP 1 were normaliβ-actin (forward: Primer 9, Table 1; reverse: Primer 10, Table 1). To confirm the accuracy and reproducibility of real-time quantitative RT-PCR, the experiment was determined in three repeats within one LightCycler run. The results for IRE-BP 1 were normalized to the housekeeping β-actin gene. The threshold cycle (Ct) values were used to quantify the target gene expression for each sample and amplification fold of IRE-BP 1 in Cr-IRE and in the susceptible strain of *Culex pipiens pallens *was calculated using the 2^-ΔΔCt ^method [22]. The real-time PCR analysis was repeated three times with independent samples.

### Semi-quantitative RT-PCR analysis of different life cycle

RT-PCR was also done with samples isolated from Cr-IRE strain egg, 1st, 2nd, 3rd and 4th instar larvae, pupa and female adult mosquitoes to confirm the expression levels at each developmental stage using the primers described above. Mosquito β-actin gene cDNA was amplified by PCR using the forward primer (Primer 11, Table 1), and the reverse primer (Primer 12, Table 1).

The PCR conditions were: 95°C for 5 min followed by 25 cycles of 95°C for 40 s, 53°C for 40 s, 72°C for 1 min with a final 10-min extension at 72°C. The IRE-BP 1 and β-actin gene PCR products were resolved by electrophoresis on 1% agarose gels. Gels were photographed using Molecular Analyst 1.4.1 (Bio-Rad, Hercules, USA) and the images were analyzed by using BandScan 5.1 software. The magnitude of IRE-BP 1 expression in Cr-IRE strain compared to the cypermethrin sensitive strain was calculated by the following formula: (RL/Rb)/(SL/Sb). RL is the band intensity of Cr-IRE strain IRE-BP 1, Rb is the band intensity of Cr-IRE strain β-actin, SL is the band intensity of susceptible strain IRE-BP 1 and Sb is the band intensity of susceptible strain β-actin.

### Construction of the expression vector

The entire coding region of IRE-BP 1 was amplified by PCR using the specific primers designed. The reverse primer was designed to remove the original stop codon and maintained the reading frame through the DNA encoding the C-terminal peptide. The forward primer: (Primer 13, Table 1), and the reverse primer: (Primer 14, Table 1). The PCR conditions were: 95°C for 5 min, followed by 32 cycles at 95°C for 1 min, 61°C for 1.5 min and 72°C for 2 min 30 s with a final 10-min extension at 72°C. The PCR product was purified from the gel following electrophoresis using a quick Gel extraction kit (Qiagen). The purified PCR product was ligated with T4 DNA ligase to the pIB/V5-His-TOPO vector (Invitrogen, Carlsbad, USA), and the ligation reaction solution was transformed into TOP10 *E. coli *competent cells (Invitrogen). Positive clones were identified by restriction analysis of recombinants with NotI and BamHI, and by PCR with specific primers and vector primers. The accuracy of the expression plasmid pIB/V5-His-TOPO IRE-BP 1 was further verified by sequencing.

### Cell culture and stable transfection

*A. albopictus *mosquito C6/36 cells were obtained from the China Center for Type Culture Collection (Wuhan, China). Cells were maintained in EMEM medium supplemented with 10% (V/V) fetal bovine serum (FBS, Sigma), 100 IU/ml penicillin and 100 μg/ml streptomycin (Invitrogen). The cells were grown in a 5% CO_2 _humidified incubator at 28°C and were plated in a six-well culture plate prior to transfection. When the cells were at 50-60% confluence, they were transfected using Cellfectin (Invitrogen) according to the manufacturer's instructions. Briefly, for each well, 2 μg DNA was added to 100 μl serum-free EMEM medium, then separately, 5 μl Cellfectin reagent was added to 100 μl serum-free medium, then the two solutions were combined, mixed gently, and incubated at room temperature for 30 min. Subsequently, 0.8 ml of serum-free medium was added to this mixture and the entire mixture was layered on to cells after washing the cells with antibiotic-free medium. The cells were incubated with the transfection mixture for 24 h in a 5% CO_2 _humidified incubator at 28°C, and the transfection medium was then replaced with 2 ml normal growth medium-containing serum. The cells were then incubated for an additional 48 h before being harvested for RT-PCR and Western blotting.

Once it had been confirmed that the cells were expressing the expected protein, stable expression cell lines were created according to the manufacturer's instructions. Briefly, a kill curve was performed to test the cell line for sensitivity to 14 μg/ml blasticidin, which can kill cells within one week. Forty-eight hours post-transfection, the transfection solution was removed and fresh medium without blasticidin was added. The cells were split at a 1:5 ratio and allowed to attach for 20 min before the selective medium was added. The medium was removed and replaced with medium containing 14 μg/ml blasticidin, and the cells were incubated at 28°C. The selecting medium was replaced every 3-4 days until clones were observed. Eight days later, the medium was replaced with medium containing 7 μg/ml blasticidin. The resistant cell lines were isolated using a dilution method until only one colony was found in each well of a 96-well microtiter plate, after which the plate was incubated until the colony filled most area of the well. The cells were harvested and transferred to a 24-well plate with 0.5 ml fresh medium containing 7 μg/ml blasticidin, then the clone was expanded in 12-and 6-well plates, and finally a T-25 flask. The cells were analyzed for expression using RT-PCR and Western blotting.

### Isolation of total RNA and RT-PCR analysis of the specific IRE-BP 1 transcript

Total RNA was isolated from transfected cells by using Trizol reagent (Invitrogen) according to the manufacturer's protocol. Five micrograms of isolated total RNA from each sample was used as a template for first-strand cDNA synthesis. The cDNA was synthesized at 70°C for 5 min, and 0°C for 5 min, then 37°C for 1.5 h with a random primer using Avian myeloblastosis virus (AMV) reverse transcriptase (TaKaRa). The reverse transcriptase was then inactivated at 99°C for 5 min. PCR amplification of the IRE-BP 1 gene was performed with the forward gene-specific primer (Primer 15, Table 1) and the reverse vector primer ((Primer 16, Table 1) for confirmation of transcriptional expression. The primers used for β-actin PCR amplification were: forward (Primer 17, Table 1) and reverse (Primer 18, Table 1). One microliter of the RT reaction product was used as the template for routine PCR. The following cycling parameters were used: 95°C for 5 min followed by 25 cycles of 95°C for 1 min, 60°C for 90 s and 72°C for 2.5 min, followed by a final extension step of 72°C for 10 min.

### Western blot analysis

Protein was extracted from transfected cells by KEYGEN Total Protein Extraction Kit (Nanjing, China) according to the manufacturer's instructions and concentrations were determined by BCA Protein Assay kit (Pierce, USA). Twenty micrograms of protein per lane was used for SDS-PAGE electrophoresis. The gels were run for 100 min at 100 V and protein was transferred to a PVDF membrane at 40 V for 30 min then blocked in TBST and 5% non-fat dry milk for 2 h. Detection was done with the diaminobenzidine (DAB) chromogenic kit (Boster BioTech Company, Wuhan, China) according to the manufacturer's instructions. The membrane was exposed in visible light for 10 min, then washed with ddH2O to terminate the reaction. Molecular weight was determined by comparison with molecular weight markers (Bio-Rad).

### ^3^H-TdR incorporation

C6/36 cells were kept in the presence of various concentrations of cypermethrin (1, 5, 10, 20, 40, 80, 120, 160 and 200 μM) for 72 h before ^3^H-TdR incorporation. Eighteen hours before harvesting, 1lCi of ^3^H-TdR was added to the medium of each well. To harvest the cells, EMEM medium was discarded, and cells were washed three times with 0.05 M PBS (pH 7.4), and detached from the microtiter wells by trypsinization. Detached C6/36 cells were harvested onto glass fiber filter paper using a mini-MASH II microharvesting device (Whittaker MA Bioproducts, Walkersville, MD) and ^3^H-TdR incorporated into C6/36 (cell associated ^3^H-TdR) was determined using a Wallac 1414 (WALLAC, Finland) liquid scintillation counter according to the manufacturer's instructions. Relative viability (%) was calculated as the ratio of ^3^H-TdR reduction in treated cells to that of control cells (C6/36 cells transfected with vector and null-transfected C6/36 cells). Each condition was performed in triplicate.

### Statistical analysis

The inhibitive effect (E) of the cypermethrin on the cell viability was described by the equation: E = (E_max_*C)/(EC_50_+C). Horizontal axis is the concentration of cypermethrin, vertical axis is the inhibition rate; Emax is the concentration at which maximum effect is reached, while EC_50 _(50% effective dose [ED_50_]) is the concentration at which 50% of the maximum effect is reached. The 95% confidence intervals were used to determine significant differences among different cells.

## Results

### Cloning of IRE-BP 1 full length cDNA

**Figure 1:**
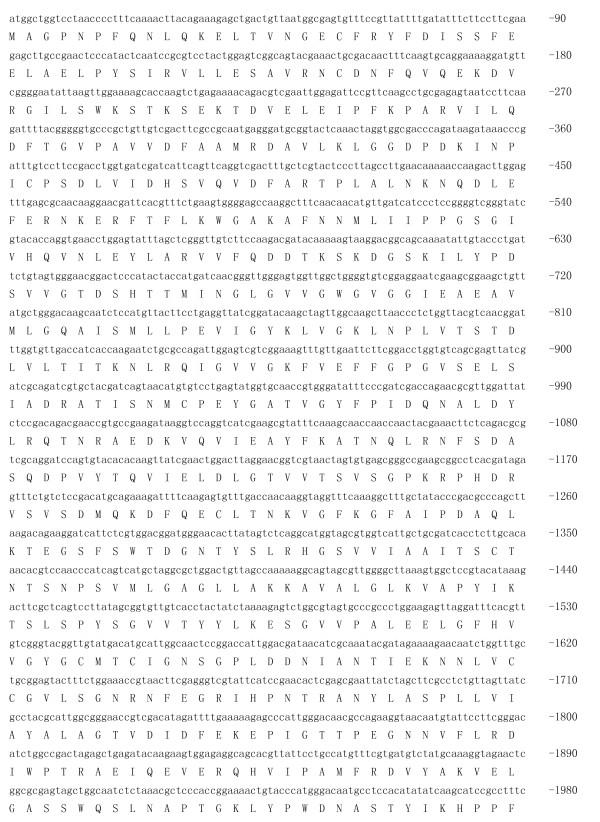
**cDNA sequence and deduced amino acid sequence of IRE-BP 1**. cDNA sequence and deduced amino acid sequence of IRE-BP 1. Stars denote the stop codon TAA. GenBank Accession No. HM443949.

**Figure 2:**
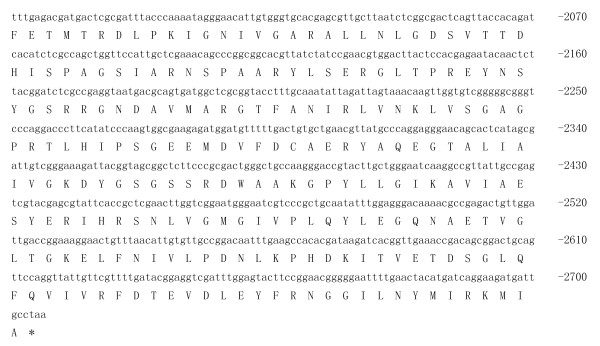
**cDNA sequence and deduced amino acid sequence of IRE-BP 1**. cDNA sequence and deduced amino acid sequence of IRE-BP 1. (continued)

**Figure 3:**
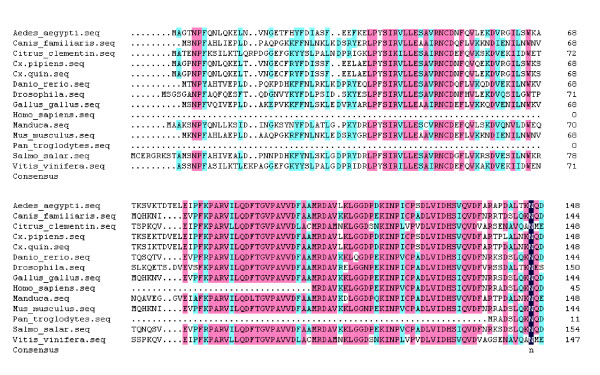
**Amino acid sequence alignment of IRE-BP 1 in different species**. Amino acid sequence alignment of IRE-BP 1 in different species. Abbreviations and GenBank Accession Numbers: *Aedes aegypti: *AY445078; *Canis familiaris: *XM_538698; *Citrus clementina: *FN552254; *Culex pipiens pallens: *HM443949; *Culex quinquefasciatus: *XM_001843334; *Danio rerio: *NM_001034983; *Drosophila melanogaster: *NM_058023; *Gallus gallus: *D16150; *Homo sapiens: *AF261088; *Manduca sexta: *AY032658; *Mus musculus: *AJ427344; *Pan troglodytes: *XM_001155874; *Salmo salar: *NM_001140230; *Vitis vinifera: *XM_002263301.

**Figure 4:**
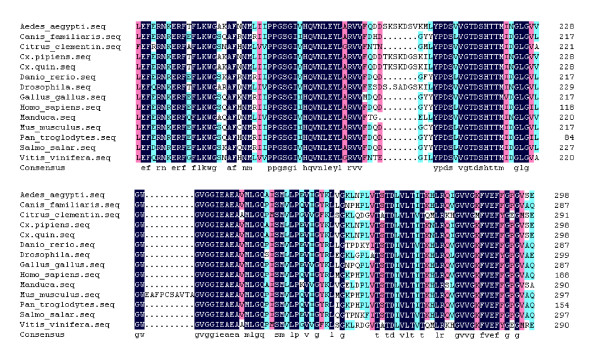
**Amino acid sequence alignment of IRE-BP 1**. Amino acid sequence alignment of IRE-BP 1 in different species. (continued)

**Figure 5:**
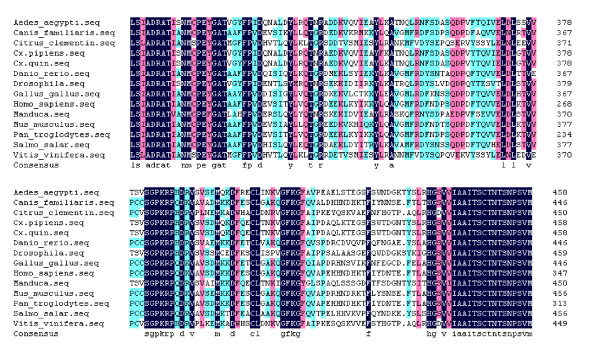
**Amino acid sequence alignment of IRE-BP 1**. Amino acid sequence alignment of IRE-BP 1 in different species. (continued)

**Figure 6:**
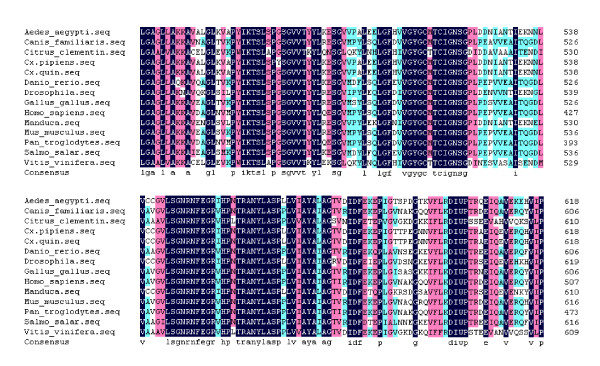
**Amino acid sequence alignment of IRE-BP 1**. Amino acid sequence alignment of IRE-BP 1 in different species. (continued)

**Figure 7:**
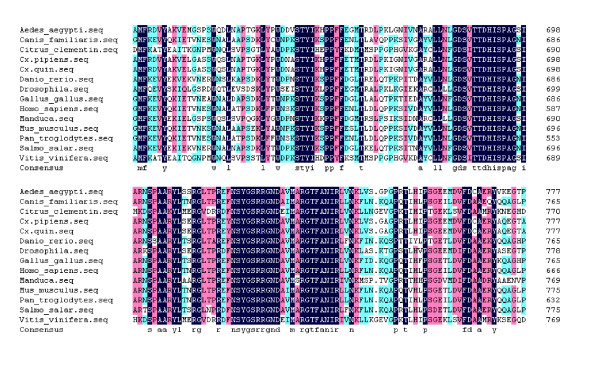
**Amino acid sequence alignment of IRE-BP 1**. Amino acid sequence alignment of IRE-BP 1 in different species. (continued)

**Figure 8:**
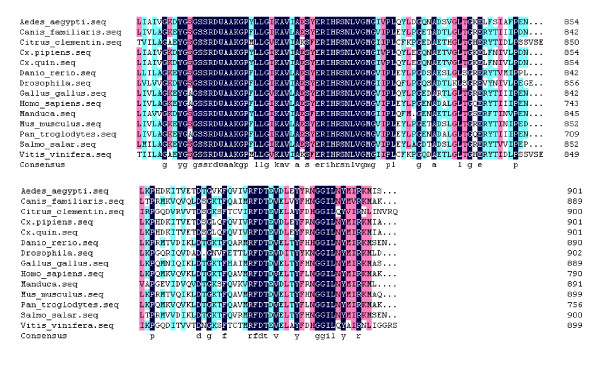
**Amino acid sequence alignment of IRE-BP 1**. Amino acid sequence alignment of IRE-BP 1 in different species. (continued)

**Figure 9:**
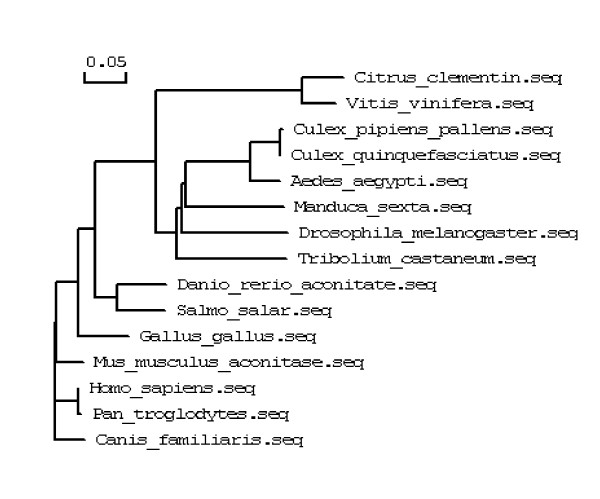
**Phylogenetic relationships of IRE-BP 1**. Phylogenetic relationships of IRE-BP 1 among *Culex pipiens pallens *and some other species. Abbreviations and GenBank Accession Numbers: *Aedes aegypti: *AY445078; *Canis familiaris: *XM_538698; *Citrus clementina: *FN552254; *Culex pipiens pallens: *HM443949; *Culex quinquefasciatus: *XM_001843334; *Danio rerio: *NM_001034983; *Drosophila melanogaster: *NM_058023; *Gallus gallus: *D16150; *Homo sapiens: *AF261088; *Manduca sexta: *AY032658; *Mus musculus: *AJ427344; *Pan troglodytes: *XM_001155874; *Salmo salar: *NM_001140230; *Tribolium castaneum: *XM_967008; *Vitis vinifera: *XM_002263301.

A fragment of the expected size (2706 bp) was cloned and sequenced. Then the full sequence was accessed by GenBank (Figure 1, 2, GenBank Accession No. HM443949). The longest open reading frame encoded 901 amino acids with a predicted molecular mass of 98.75 kDa. The predicted isoelectric point is 5.54 by ExPASy. Amino acid sequence alignment of IRE-BP 1 was conducted by ClustalW software in different species (Figure 3, 4, 5, 6, 7, 8). Phylogenetic relationships of IRE-BP 1 among *Cx. pipiens pallens *and some other species showed IRE-BP 1 of *Cx. pipiens pallens *has the highest homology with *Culex quinquefasciatus *IRE-BP 1 g (Figure 9). SMART showed there is a typical IRE-BP 1 domain found. SIGNAL P3.0 indicted there that no peptide sequence existed in the sequence.

### Real-time quantitative RT-PCR analysis

**Figure 10:**
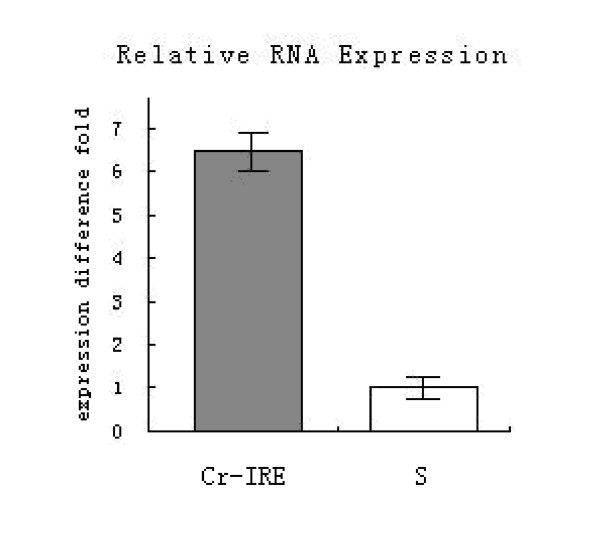
**Quantitative RT-PCR assay of IRE-BP 1**. Quantitative RT-PCR assay of IRE-BP 1 mRNA in Cr-IRE and susceptible strains of *Culex pipiens pallens*. Quantitative RT-PCR was performed by using a Lightcycle-RNA amplification Kit SYBR Green and repeated using three independently purified RNA samples. The enhancement of fluorescence was found to be proportional to the initial concentration of template cDNA. IRE-BP 1 transcript copy numbers were normalized based on expression of the housekeeping b-actin from respective strains. Cr-IRE represents Cr-IRE strain and S represents susceptible strain. The data are presented as means ± SD. n = 3, p = 0.03.

The IRE-BP 1 exhibited 6.7-fold higher level of transcription in the Cr-IRE strain than in the susceptible strain. The results suggested that IRE-BP 1 expression was up-regulated in the Cr-IRE strain (Figure 10).

### Expression of IRE-BP 1 gene at various developmental stages in Cr-IRE and susceptible *Cx. pipiens pallens*

**Figure 11:**
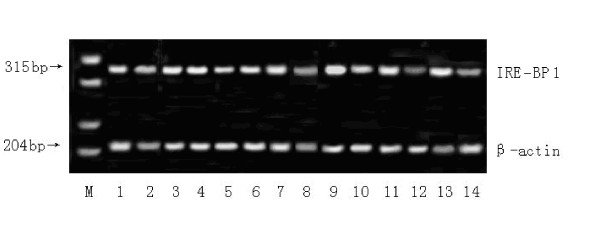
**Semi-quantitative RT-PCR**. Semi-quantitative RT-PCR of each life stage showed the relative amount of amplified transcripts of IRE-BP 1 (panel 1) in comparison with amplified mosquito b-actin gene transcripts (panel 2). M, marker; 1, Cr-IRE strain egg; 2, susceptible strain egg; 3, Cr-IRE strain 1st larvae; 4, susceptible strain 1st larvae; 5, Cr-IRE strain 2nd larvae; 6, susceptible strain 2nd larvae; 7, Cr-IRE strain 3rd larvae; 8, susceptible strain 3rd larvae; 9, Cr-IRE strain 4th larvae; 10, susceptible strain 4th larvae; 11, Cr-IRE strain pupa; 12, susceptible strain pupa; 11, Cr-IRE strain pupa; 12, susceptible strain pupa; 13, Cr-IRE strain female mosquito; 14, susceptible strain female mosquito.

A 315-bp cDNA fragment was selectively amplified using the specific primers. The amount of amplified cDNA product was normalized by comparison with the amplification product of the β-actin gene from *Cx. pipiens pallens *(204 bp). The maximal level of IRE-BP 1 mRNA was detected in adult female mosquito, followed by the 4th instar larvae and the pupa. In all of the developmental stages, Cr-IRE strain has 3-to 4-fold increase of IRE-BP 1 transcription compared to the susceptible strain (Figure 11).

### Transcription and expression of IRE-BP 1 in C6/36 cells

**Figure 12:**
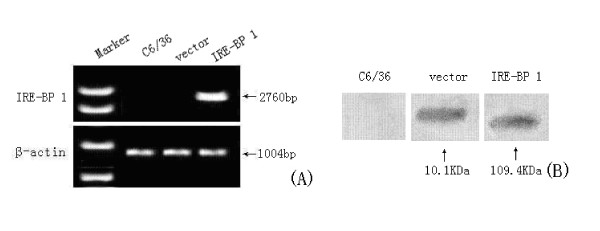
**Effects of expression and transcripts of IRE-BP 1 in cells**. (A) RT-PCR analysis of IRE-BP 1 mRNA in mosquito cells using forward primer of IRE-BP 1 and reverse primer of vector. The production of the transcripts was detected in cells transfected with IRE-BP 1 (lane 2); No signal was detected either in normal cells (lane 3) or in cells transfected with vector (lane 1). (B). Western blot analysis. Expression of IRE-BP 1 was detected in cells transfected with IRE-BP 1 (lane 3), expression of vector protein was detected in cells transfected with vector (lane 2), no signal was detected in normal cells (lane 1).

A PCR product of the expected size, about 2760 bp, was observed only in cells that were transfected with the IRE-BP 1 gene (Figure 12A), confirming that IRE-BP 1 had been transcribed in the transfected cells. Western blot analysis using anti-His antibodies identified a protein of 109.4 kDa molecular weight in cells transfected with the IRE-BP 1 gene (Figure 12B), confirming the exogenous IRE-BP 1 was expressed in C6/36 cells successfully.

### Viability assay

**Figure 13:**
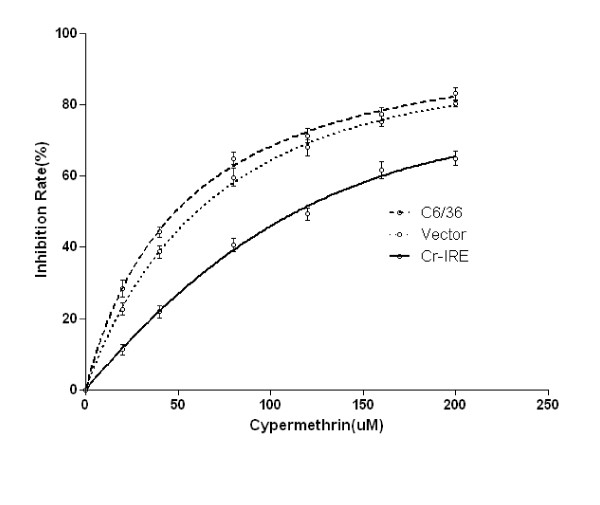
**Inhibitive effect of cypermethrin on cell viability**. The cultured cells were treated with different concentration of cypermethrin and incubated for 72 h. The inhibitive effect(E)of the cypermethrin on the cell viability was described by the equation: E = (Emax*C)/(EC_50 _+ C).

Expression of the IRE-BP 1 gene improved the viability of cypermethrin-treated C6/36 cells. The EC_50 _and 95% confidence intervals of null-transfected cells are 35.24 (31.88-40.17), the EC_50 _and 95% confidence intervals of vector-transfected cells are 42.31 (34.19-49.26), and the EC_50 _and 95% confidence intervals of IRE-BP 1 transfected cells are 101.34 (91.23-113.71). Obvious cell viability augmentation was observed in the IRE-BP 1 transfected C6/36 cells compared to null-transfected or vector-transfected cells (Figure 13).

## Discussion

Insecticide resistance has become a serious public health problem, which limited the effectiveness of pest control and presented an obstacle to the control of vector borne diseases [12,23,24]. Here we report the characterization of one cypermethrin resistance associated gene of *Cx. pipiens pallens *[25]. The writer reported insecticide-resistance ribosomal protein L39 gene of *Cx. pipiens pallens*, while other researchers in the same laboratory identified several genes differentially expressed in cypermethrin-resistant strain of the mosquito previously [15,26-37]. Some of the candidate genes encode for enzymes such as chymotrypsin [30], trypsin [34], CYP450 [29], which are likely involved in more direct functions, e.g. oxidation of cypermethrin, others are likely more upstream factors, such as ribosomal protein L39 [25]. The predicted *Culex pipiens pallens *IRE-BP 1 protein has many characteristics common to the IRE-BP 1 family, and the *Cx. pipiens pallens *IRE-BP 1 protein sequence shared 99.45% identity with that of *Cx. quinquefasiatus*. These results strongly suggest that this protein is the IRE-BP 1 of *Cx. pipiens pallens*.

We determined the expression profile of IRE-BP 1 mRNA during the mosquito life cycle. Our results demonstrated that the levels of IRE-BP 1 expression are developmentally regulated, and the expression was the highest in adult mosquitos.

IRE-BP binds to iron-responsive elements (IREs), which were first identified in the 5' UTR of ferritin mRNA and in the 3'UTR of transferrin receptor (TftR) mRNA. Binding of IRE-BP to IREs is regulated by the iron status of the cell. Iron starvation activates binding and thus represses ferritin mRNA translation and stabilizes TfR transcripts *in vivo*. The effect of changes in iron availability *in vivo *can be mimicked by alterations in the redox environment of IRE-BP *in vitro*. This observation has led to the suggestion that IRE-BP activity is regulated post-translationally by the reversible oxidation-reduction of cysteinyl sulthydryl groups important for the interaction of IRE-BP and IREs.

In cells that are iron-depleted, the Fe-S cluster of cytosolic aconitase is disrupted, resulting in a loss of aconitase activity and the exposure of the RNA-binding site so that, as the IRE-BP, it can bind with high affinity to the IRE [19]. In the presence of adequate iron, the [4Fe-4S] cluster is reassembled, enzyme activity is regained, and RNA binding is lost. The binding of the IRE-BP to the IRE found in the 5' untranslated region of transcripts for ferritin or the erythroid form of 5-aminolevulinate synthase down-regulates protein synthesis, most likely through an inhibition of translation initiation [38]. In the case of transferrin receptor regulation, five IREs are found within the 3' untranslated region of the transcript and their interaction with the IRE-BP results in a stimulation of transferrin receptor synthesis by inhibiting degradation of the mRNA [9]. The dynamic properties of the Fe-S cluster in the IRE-BP/cytosolic aconitase suggested that it might serve as a target for NO produced in response to glutamatergic stimulation in neurons. No association between IRE-BP 1 and pyrethroid resistance was established before. In this study, we found a higher transcriptional level of IRE-BP 1 in the Cr-IRE strain than in the susceptible strain. Ectopic expression of IRE-BP 1 in C3/36 cells also increases resistance against cypermethrin. The up-regulation of IRE-BP 1 in adult mosquito may be indicative of an adaptive ability of the mosquito to regulate the synthesis of other insecticide resistance proteins and consequently to have enhanced resistance to insecticides. Although we do not have enough information to pinpoint the exact role of IRE-BP 1 in cypermethrin resistance, our results suggest that IRE-BP 1 is a good candidate for future studies of pyrethroid resistance.

The IRE-BP 1 family spans both vertebrates and invertebrates. It includes serum IRE-BP 1, ovo IRE-BP 1, lactoferrin, melano IRE-BP 1, inhibitor of carbonic anhydrase, saxiphilin, the major yolk protein in sea urchins, the crayfish protein, pacifastin, and a protein from green algae. Most (but not all) contain two domains of around 340 residues, thought to have evolved from an ancient duplication event. For serum IRE-BP 1, ovo IRE-BP 1 and lactoferrin each of the duplicated lobes binds one atom of Fe (III) and one carbonate anion. With a few notable exceptions each iron atom is coordinated to four conserved amino acid residues: an aspartic acid, two tyrosines, and a histidine, while anion binding is associated with an arginine and a threonine in close proximity. These six residues in each lobe were examined for their evolutionary conservation in the homologous N- and C-lobes of 82 complete IRE-BP 1 sequences from 61 different species. Of the ligands in the N-lobe, the histidine ligand shows the most variability in sequence. Also, of note, four of the twelve insect IRE-BP 1s have glutamic acid substituted for aspartic acid in the N-lobe (as seen in the bacterial ferric binding proteins). In addition, there is a wide spread substitution of lysine for the anion binding arginine in the N-lobe in many organisms including all of the fish, the sea squirt and many of the unusual family members i.e., saxiphilin and the green alga protein. It is hoped that this short analysis will provide the impetus to establish the true function of some of the TF family members that clearly lack the ability to bind iron in one or both lobes and additionally clarify the evolutionary history of this important family of proteins.

## Conclusions

In summary, we have cloned the first IRE-BP 1 gene known from *Cx. pipiens pallens*. Based on the characteristics of the gene, it is a member of the IRE-BP 1 family. The data in the present study suggests that IRE-BP 1 may confer some cypermethrin resistance in mosquitoes. Research carried out to date has provided a basis for further studies on the gene function associated with insecticide resistance, which will improve our understanding of the molecular basis of IRE-BP 1 mediated resistance in *Cx. pipiens pallens*.

## Competing interests

The authors declare that they have no competing interests.

## Authors' contributions

Wenbin Tan coordinated and carried out the whole experiment and manuscript writing, Xiao Wang participated in the cell culture and data analysis. Peng Cheng participated in the sequence alignment. Lijuan Liu and Haifang Wang participated in the sequence alignment. Maoqing Gong participated in the RT-PCR and RACE experiment. Xin Quan participated in the ^3^H-TdR experiment. Honggang Gao participated in the Real-time quantitative RT-PCR analysis. Changliang Zhu participated in the design of the study and performed the statistical analysis.

All authors read and approved the final manuscript.
